# Analysis of diagnostic characteristics and clinical related factors of 70 patients with atelectasis by painless bronchoscopy

**DOI:** 10.12669/pjms.38.6.5273

**Published:** 2022

**Authors:** Qiang Li, Jun Sun, Xuefen Shuai, Junqing Ren, Xuedong Chen

**Affiliations:** 1 Qiang Li, Department of Respiratory and Critical Medicine, Xuancheng people’s Hospital, Xuancheng 242000, Anhui, China; 2 Jun Sun, Department of Respiratory and Critical Medicine, Xuancheng people’s Hospital, Xuancheng 242000, Anhui, China; 3 Xuefen Shuai, Department of Respiratory and Critical Medicine, Xuancheng people’s Hospital, Xuancheng 242000, Anhui, China; 4 Junqing Ren, Department of Respiratory and Critical Medicine, Xuancheng people’s Hospital, Xuancheng 242000, Anhui, China; 5 Xuedong Chen, Department of Respiratory and Critical Medicine, Xuancheng people’s Hospital, Xuancheng 242000, Anhui, China

**Keywords:** Pulmonary atelectasis, Bronchoscopy, Diagnosis, Etiology

## Abstract

**Objectives:**

To analyze the diagnostic characteristics and clinical related factors of patients with atelectasis by painless electronic bronchoscopy.

**Methods:**

A retrospective analysis was performed on 70 patients with atelectasis admitted to Xuancheng People’s Hospital from April 2019 to June 2021. The clinical data of the patients and the diagnosis characteristics under painless electronic bronchoscope were analyzed, and the clinical related factors were investigated.

**Results:**

Seventy patients with atelectasis underwent pathological examination and bacteriological examination after painless electronic bronchoscopy, including 16 cases (22.86%) of inflammation, 11 cases (15.71%) of tuberculosis, 36 cases (51.43%) of tumor, one case (1.43%) of inflammatory polyp, one case (1.43%) of congenital dysplasia, two cases (2.86%) of foreign body inhalation, and three cases (4.29%) of other symptoms. Male patients with atelectasis showed most cauliflower-like tumors and mucosal swelling under electronic bronchoscopy (P<0.05), while female patients showed scar occlusion/stenosis at most (P<0.05). Middle-aged and elderly patients under electronic bronchoscopy showed most cauliflower-like tumors, scar stenosis/occlusion and mucosal swelling cavity, while young patients mainly showed necrosis and purulent secretions. Heavy smokers were most likely to have cauliflower-like tumors, while non-smokers were predominantly with scar stenosis/occlusion.

**Conclusions:**

Painless electronic bronchoscopy is of great value in the clinical diagnosis of patients with atelectasis, and it is likely to further clarify the etiology of atelectasis. Age, sex, and quantity of smoking may be clinical factors associated with atelectasis.

## INTRODUCTION

Atelectasis, is a common respiratory disease in clinical practice, is a kind of apneumatosis or reduced lung capacity caused by a variety of reasons associated with collapse of lung tissue and reduced lung volume.^1^ It is often a comprehensive manifestation of a variety of diseases rather than an independent disease.^2^,^3^ In recent years, with the change of environment, climate and aging population, the etiology of atelectasis has changed to some extent, but lung cancer, tuberculosis and lung inflammation have always been the main causes of atelectasis.^4^,^5^ Imaging examinations and physical examination findings are currently the first preferred diagnostic means for atelectasis, while conventional imaging examination has no obvious specificity and can only determine the site of the disease rather than the etiology.^6^ Therefore, adverse consequences, such as delay of treatment time and influence of prognosis of patients, often occur if a clear diagnosis cannot be given in time clinically.^7^ Compared with fiberoptic bronchoscopy, electronic bronchoscopy has been widely used in the diagnosis and treatment of atelectasis in recent years because of its lighter material, less trauma and more convenient operation. Painless electronic bronchoscopy was favored by patients because the use of anesthetics has the advantages of less pain.^8^,^9^ This study retrospectively analyzed the clinical data of 70 patients with atelectasis, aiming to explore the diagnostic characteristics and clinical related factors of painless electronic bronchoscopy in the treatment of atelectasis.

## METHODS

A total of 70 patients with atelectasis admitted to Xuancheng People’s Hospital from April 2019 to June 2021 were recruited, including 51 males and 19 females, aged from 18 to 79 years, with an average age of 43.23±19.67 years. All patients were divided into three groups according to the standard of age < 40 years old, 40-60 years old, and > 60 years old: young age group, middle-aged group and elderly group, with 4 cases in the young age group, 16 cases in the middle-aged group and 50 cases in the elderly group. All the patients were admitted for clinical symptoms, including 51 cases of cough (72.86%), 28 cases of hemoptysis (40.00%), 17 cases of chest pain (24.29%), 16 cases of shortness of breath (22.86%) and 3 cases of fever (4.29%). The smoking status of the patients was as follows: 34 cases of non-smokers and 36 cases of smokers. Smoking patients were divided into light smokers (< 400 cigarettes per year) (11 cases) and heavy smokers (≥400 cigarettes per year) (25 cases) according to the amount of smoking.

### Ethical Approval

The study was approved by the Institutional Ethics Committee of Xuancheng people’s Hospital on June 16, 2019(No.:2017020), and written informed consent was obtained from all participants.

### Inclusion criteria:

• Patients diagnosed with atelectasis by chest CT;
• Patients with clinical symptoms such as cough, expectoration, hemoptysis, chest distress, chest pain, fever, wheezing, shortness of breath, fatigue and emaciation;
• Patients who gave informed consent and signed informed consent for this study.

### Exclusion criteria:

• Patients with severe cardiovascular diseases and liver and kidney diseases;
• Patients with severe rheumatic immune system diseases and hematological malignancies;
• Patients who are unable to tolerate painless electronic bronchoscopy.

### Examination Methods

All patients were fasted for 8 hours and water was forbidden for 6 hours before examination. Painless electronic bronchoscopy was performed for all patients. 30min prior to examination, the patients were in supine position, then given intramuscular injection of 0.5mg atropine (Anhui Changjiang Pharmaceutical Co., LTD., State Drug Approval No.: H34021900, chemical drug, 1ml:0.5mg) to reduce respiratory secretions, and removable dentures were removed. And five minutes before operation, nasal and oropharyngeal surface anesthesia was performed with 10 ml of 2% lidocaine (Shanghai Hefeng Pharmaceutical Co., LTD., State Drug Approval No.: H20023777, chemical drug, 5ml:0.1g). Patients were treated with 3ml 2% lidocaine sprayed down the throat in several doses, followed by inhalation of an appropriate amount of 2% lidocaine (3-5ml) by aerosol inhalation. Then an intravenous channel was established for the patient. After successful superficial anesthesia, the patients were given 2mg of midazolam (Jiangsu Nhwa Pharmaceutical Co., Ltd., State Drug Approval No.: H10980025, chemical drug, 2ml:10mg), 10mg of etomide (Jiangsu Nhwa Pharmaceutical Co.,Ltd, National drug Approval H20020511, Chemical drug, 10ml:20mg), 35μg of sufentanil citrate (Yichang Renfu Pharmaceutical Co., LTD., State Drug Approval No.: H20054171, chemical drug, 1ml:50μg(by sufentanil)) and propofol (Xi’an Liban Pharmaceutical Co., LTD., State Drug Approval No.: H19990282, chemical drug, 20ml:0.2g) via the intravenous by an anaesthetist. After sedation, laryngeal mask ventilation was given, and the electronic bronchoscope was slowly inserted into the trachea along the oral cavity and through the laryngeal mask. Glottis, trachea, bronchus, lobes, segments and subsegments of bronchus were examined. The lesion sites were observed with the focus of CT scanning for biopsy, and the samples were sent for pathological examination and bacteriological examination. BF-P150 electronic bronchoscope and BF-T150 electronic bronchoscope produced by Olympus Company of Japan were used as the examination bronchoscope in this study.

### Statistical Method

SPSS20.0 software was utilized to process the experimental results of this study. *X^2^* test for R×C table was adopted for the comparison of multiple composition ratios, while *X^2^* test of four-lattice table was used for the comparison of the two sample rates. Set α=0.05, *P<*0.05 indicates a significant difference.

## RESULTS

Seventy patients with atelectasis underwent pathological examination and bacteriological examination after painless electronic bronchoscopy, including 16 cases (22.86%) of inflammation, 11 cases (15.71%) of tuberculosis, 36 cases (51.43%) of tumor, one case (1.43%) of inflammatory polyp, one case (1.43%) of congenital dysplasia, two cases (2.86%) of foreign body inhalation, and 3 cases (4.29%) of other symptoms. As can be seen from Table-I, the majority of patients with cauliflower-like tumors under bronchoscopy had tumors (93.75%), among which squamous cell carcinoma was the most common (68.75%), tuberculosis was the most common (33.33%) among the patients presenting as scar stenosis/occlusion, and inflammation was the most common (38.46%) among the patients presenting as purulent or bloody secretions. Moreover, various etiologies can be manifested as mucosal swelling, unevenness or necrosis under electronic bronchoscopy.

**Table I:** Distribution of bronchoscopic manifestations and etiology in patients with atelectasis (X±s).

Group	Squamous cell carcinoma	Adenocarcinoma	Small cell carcinoma	Inflammation	Tuberculosis	Other	Total
Cauliflower-like tumor	11 (68.75%)	2 (12.50%)	2 (12.50%)	0 (0.00%)	0 (0.00%)	1 (6.25%)	16
Scar stenosis/occlusion	2 (22.22%)	2 (22.22%)	0 (0.00%)	0 (0.00%)	3 (33.33%)	2 (22.22%)	9
Mucosal swelling	2 (13.33%)	1 (6.67%)	4 (26.66%)	7 (46.67%)	1 (6.67%)	0 (0.00%)	15
Necrosis	1 (11.11%)	1 (11.11%)	1 (11.11%)	2 (22.22%)	3 (33.33%)	1 (11.11%)	9
Purulent discharge	1 (12.50%)	1 (12.50%)	1 (11.11%)	4 (50.00%)	1 (12.50%)	0 (0.00%)	8
Bloody secretions	1 (20.00%)	1 (20.00%)	0 (0.00%)	1 (20.00%)	1 (20.00%)	1 (20.00%)	5
Tracheal hardening	0 (0.00%)	0 (0.00%)	0 (0.00%)	1 (50.00%)	1 (50.00%)	0 (0.00%)	2
Almost normal	1 (25.00%)	1 (25.00%)	0 (0.00%)	1 (25.00%)	1 (25.00%)	0 (0.00%)	4
Foreign body	0 (0.00%)	0 (0.00%)	0 (0.00%)	0 (0.00%)	0 (0.00%)	2 (100.00%)	2

Total	19	9	8	16	11	7	70

Male patients with atelectasis showed most cauliflower-like tumors and mucosal swelling under electronic bronchoscopy (P<0.05), while female patients showed scar occlusion/stenosis at most (P<0.05); Table-II, Middle-aged and elderly patients under electronic bronchoscopy showed most cauliflower-like tumors, scar stenosis/occlusion and mucosal swelling cavity, while young patients mainly showed necrosis and purulent secretions, P<0.05 indicates a statistically significant difference.Table-III The electronic bronchoscopy manifestations of atelectasis patients with different smoking degrees also showed differences in composition. Among them, heavy smokers were most likely to have cauliflower-like tumors, while non-smokers were predominantly with scar stenosis/occlusion, P<0.05 indicates a statistically significant difference. Table-IV

**Table II:** Electronic bronchoscopic manifestations of patients with atelectasis of different genders (n, %).

Group	Cauliflower-like tumor	Scar stenosis/occlusion	Mucosal swelling	Necrosis	Purulent discharge	Bloody secretions	Tracheal hardening	Almost normal	Foreign body
Male (n=51)	14 (27.45%)	2 (3.92%)	14 (27.45%)	6 (11.76%)	5 (9.80%)	4 (7.84%)	1 (1.96%)	3 (5.9%)	2 (3.92%)
Female (n=19)	2 (10.53%)	7 (36.84%)	1 (5.26%)	3 (15.79%)	3 (15.79%)	1 (5.26%)	1 (5.26%)	1 (5.26%)	0 (0.00%)
X^2^	2.2488	13.3901	4.0476	0.2001	0.4899	0.1389	0.5439	0.1389	0.3779
P	<0.05	<0.05	<0.05	>0.05	>0.05	>0.05	>0.05	>0.05	>0.05

**Table III:** Electronic bronchoscopic manifestations of atelectasis patients of different ages (n, %).

Group	Cauliflower-like tumor	Scar stenosis/occlusion	Mucosal swelling	Necrosis	Purulent discharge	Bloody secretions	Tracheal hardening	Almost normal	Foreign body
Young age group (n=4)	0 (0.00%)	0 (0.00%)	0 (0.00%)	2 (50.00%)	2 (50.00%)	0 (0.00%)	0 (0.00%)	0 (0.00%)	0 (0.00%)
Middle-aged group (n=16)	5 (31.25%)	4 (25.00%)	3 (18.75%)	1 (6.25%)	0 (0.00%)	1 (6.25%)	1 (6.25%)	1 (6.25%)	0 (0.00%)
Elderly group (n=50)	11 (22.00%)	5 (10.00%)	12 (24.00%)	6 (12.00%)	6 (12.00%)	4 (8.00%)	1 (2.00%)	3 (6.00%)	2 (4.00%)
X^2^	1.4326	1.2750	0.6873	5.4935	6.2353	0.1938	0.4632	0.938	0.4058
P	<0.05	>0.05	>0.05	<0.05	<0.05	>0.05	>0.05	>0.05	>0.05

**Table IV:** Electronic bronchoscopic manifestations of patients with atelectasis of different smoking levels (n, %).

Group	Cauliflower-like tumor	Scar stenosis/occlusion	Mucosal swelling	Necrosis	Purulent discharge	Bloody secretions	Tracheal hardening	Almost normal	Foreign body
Non-smoking group (n=34)	3 (8.82%)	7 (20.59%)	6 (17.65%)	5 (14.71%)	5 (14.71%)	3 (8.82%)	1 (2.94%)	2 (5.88%)	2 (5.88%)
Light smoking group (n=11)	2 (18.18%)	1 (9.09%)	3 (27.27%)	1 (9.09%)	1 (9.09%)	1 (9.09%)	1 (9.09%)	1 (9.09%)	0 (0.00%)
Heavy smoking group (n=25)	11 (44.00%)	1 (4.00%)	6 (24.00%)	3 (12.00%)	2 (8.00%)	1 (4.00%)	0 (0.00%)	1 (4.00%)	0 (0.00%)
X^2^	9.8500	3.3823	0.3589	0.0900	0.6196	0.5304	0.7410	0.5304	0.7480
P	<0.05	<0.05	>0.05	<0.05	>0.05	>0.05	>0.05	>0.05	>0.05

## DISCUSSION

Atelectasis is a disease of reduced lung volume and collapse of lung tissue caused by apneumatosis or reduced lung capacity, which can be mainly attributed to bronchial obstruction, among which the bronchial obstruction is the most common.^10^,^11^ The main substances that cause bronchial obstruction are tumors, blood clots, tuberculosis, secretions and foreign bodies, etc., with a large proportion of tumors^12^, which may be closely related to environmental factors. Patients with atelectasis are often clinically presented with cough, hemoptysis, chest tightness, fever, shortness of breath, wheezing, etc.^13^,^14^ CT examination is one of the effective methods for diagnosing atelectasis, which is characterized by the advantages of identifying the location of the disease and discovering the pathological changes of surrounding organs.^15^ However, it is worth noting that CT examination fails to identify the etiology of atelectasis in patients to help them with timely and targeted treatment.^16^ In the wake of the widespread application of painless electronic bronchoscopy technology in the diagnosis of atelectasis, more and more attention has been paid to the diagnostic characteristics and advantages of painless electronic bronchoscopy, which boasts a variety of advantages such as pain relief, improved compliance, shorter examination times, diminished injuries, reduced risk of intraoperative complications and increased positive examination rates.^17^,^18^ Painless electronic bronchoscopy can accurately locate the lesion area, observe the obstruction in the bronchial lumen and the morphological changes of atelectasis, and determine the etiology by virtue of further cytological, pathological and bacteriological examinations.^19^,^20^

According to the data of this study, patients with atelectasis underwent pathological examination and bacteriological examination after painless electronic bronchoscopy, including 16 cases (22.86%) of inflammation, 11 cases (15.71%) of tuberculosis, 36 cases (51.43%) of tumor, one case (1.43%) of inflammatory polyp, one case (1.43%) of congenital dysplasia, two cases (2.86%) of foreign body inhalation, and three cases (4.29%) of other symptoms. The majority of patients with cauliflower-like tumors under bronchoscopy had tumors (93.75%), among which squamous cell carcinoma was the most common (68.75%), tuberculosis was the most common (33.33%) among the patients presenting as scar stenosis/occlusion, and inflammation was the most common (38.46%) among the patients presenting as purulent or bloody secretions. Moreover, various etiologies can be manifested as mucosal swelling, unevenness or necrosis under electronic bronchoscopy; Male patients with atelectasis showed most cauliflower-like tumors and mucosal swelling under electronic bronchoscopy (P<0.05), while female patients showed scar occlusion/stenosis at most (P<0.05); Middle-aged and elderly patients under electronic bronchoscopy showed most cauliflower-like tumors, scar stenosis/occlusion and mucosal swelling cavity, while young patients mainly showed necrosis and purulent secretions; Heavy smokers were most likely to have cauliflower-like tumors, while non-smokers were predominantly with scar stenosis/occlusion. P<0.05 indicates a statistically significant difference.

### Limitations of this study

It was a retrospective descriptive study, with limited clinical data available and limited persuasive conclusions. Further intervention trials are needed in the future to confirm these results.

## CONCLUSION

Painless electronic bronchoscopy is of great value in the clinical diagnosis of patients with atelectasis, and it is likely to further clarify the etiology of atelectasis. Age, sex, and quantity of smoking may be clinical factors associated with atelectasis. In order to further improve the diagnostic effect of painless electronic bronchoscopy in patients with atelectasis, the following steps should be followed: First of all, adequate attention should be given to biopsy, and the visual field should be kept clean and the material should be taken deep in the necrotic area; Secondly, brush biopsy should be fully carried out; Finally, painless bronchoscopy is imperative for patients, which contributes to improving the diagnosis rate.

### Authors’ Contributions:

**QL & JS:** Designed this study, prepared this manuscript, are responsible and accountable for the accuracy and integrity of the work;

**XC:** Collected and analyzed clinical data.

**XS & JR:** Data analysis, significantly revised this manuscript.
